# An analysis of the dust deposition on solar photovoltaic modules

**DOI:** 10.1007/s11356-018-1847-z

**Published:** 2018-03-29

**Authors:** Katarzyna Styszko, Marek Jaszczur, Janusz Teneta, Qusay Hassan, Paulina Burzyńska, Ewelina Marcinek, Natalia Łopian, Lucyna Samek

**Affiliations:** 1 Faculty of Energy and Fuels, AGH University of Science and Technology, Kraków, Poland; 2 Faculty of Electrical Engineering, Automatics, Computer Science and Biomedical Engineering, AGH University of Science and Technology, Kraków, Poland; 3 Department of Mechanical Engineering, University of Diyala, Baqubah, Iraq; 4 Faculty of Physics and Applied Computer Science, AGH University of Science and Technology, Kraków, Poland

**Keywords:** Dust accumulation, Particle deposition, Air pollution, Photovoltaic modules

## Abstract

Solid particles impair the performance of the photovoltaic (PV) modules. This results in power losses which lower the efficiency of the system as well as the increases of temperature which additionally decreases the performance and lifetime. The deposited dust chemical composition, concentration and formation of a dust layer on the PV surface differ significantly in reference to time and location. In this study, an evaluation of dust deposition on the PV front cover glass during the non-heating season in one of the most polluted European cities, Kraków, was performed. The time-dependent particle deposition and its correlation to the air pollution with particulate matter were analysed. Dust deposited on several identical PV modules during variable exposure periods (from 1 day up to 1 week) and the samples of total suspended particles (TSP) on quartz fibre filters using a low volume sampler were collected during the non-heating season in the period of 5 weeks. The concentration of TSP in the study period ranged between 12.5 and 60.05 μg m^−3^ while the concentration of PM10 observed in the Voivodeship Inspectorate of Environmental Protection traffic station, located 1.2 km from the TSP sampler, ranged from 14 to 47 μg m^−3^. It was revealed that dust deposition density on a PV surface ranged from 7.5 to 42.1 mg m^−2^ for exposure periods of 1 day while the measured weekly dust deposition densities ranged from 25.8 to 277.0 mg m^−2^. The precipitation volume and its intensity as well as humidity significantly influence the deposited dust. The rate of dust accumulation reaches approximately 40 mg m^−2^day^−1^ in the no-precipitation period and it was at least two times higher than fluxes calculated on the basis of PM10 and TSP concentrations which suggest that additional forces such as electrostatic forces significantly influence dust deposition.

## Introduction

The solar energy incident on the Earth’s surface during an hour is almost equal to the one-year total consumption on Earth. When the solar radiation penetrates the atmosphere, a significant amount of its energy is lost due to the fact that Sun radiation is absorbed by solid particles and droplets in the atmosphere and reflected by water vapour and air molecules. But the solar radiation is also absorbed by dust and another type of pollutants as well as scattered backwards, decreasing the direct solar radiation component and causing an increase of the diffuse component. For this reason, the urban and polluted areas typically receive less of the total solar radiation in reference to the clean air of the countryside or industry-free rural areas (Darwish et al. 2018; Robaa 2004). Among the varied practical applications of renewable energy, photovoltaic modules received much attention in electrical power generation and became widely used because of their low production and low maintenance cost as well as their relatively good efficiency of energy conversion which nowadays reaches 26.3% for a single-junction terrestrial cell made of silicon crystalline cell and 38.8% in the case of a five-junction silicon crystalline cell (Green et al. 2011).

However, all the types of performance of photovoltaic modules are influenced by a large number of environmental parameters (air temperature, wind speed, air pollution, the angle of incident irradiation, solar radiation spectrum, ageing, snow, dirt and shadowing) (Hassan et al. 2016; Kazem and Chaichan 2016). One of the parameters that influence the energy conversion in PV modules which can be significant in many regions is accumulated dust (Chaichan et al. 2015). The accumulated dust decreases the conversion efficiency because the dust particles reduce and scatter the intensity of the total radiation incident on the PV modules. It was revealed that the dust accumulation rate depends on several parameters such as airborne particle concentration, weather conditions, particle size distribution or type, density, shape, composition, chemistry, charge and variability in reference to the exposure time (Javed et al. 2017). The dust accumulation rate reported in literature varied 1–50 mg m^−2^ day^−1^ in Colorado (Boyle et al. 2015) and 150–300 mg m^−2^·day^−1^ in Minia, Egypt (Hegazy 2001). In another research work, a dust accumulation rate of 132 mg m^−2^·day^−1^ was reported in Mesa, Arizona (Boppana et al. 2015). The physicochemical properties of deposited dust (Kaldellis and Kapsali 2011), as well as particle size distribution of dust deposited on the surface (Said 1990), have a significant impact on the degradation of PV module performance. The size of deposited solid particles plays a major role in the scattering and absorption of radiation incident on the solar module and causes degradation of PV module efficiency. Larger particles have a greater tendency of resuspension with the airflow which promotes deposition of small-size particles. Fine particles have a greater specific surface area and generate a larger effect on cell performance degradation than a large particle with an equal mass of deposited dust (Weber et al. 2014). Research conducted by McTainsch et al. (McTainsh et al. 1997) demonstrated that the size of the dust accumulated at the surface can be divided into three ranges: small particles (a diameter of up to 5 μm) which come from widely-spaced areas, medium-size particles (20–40 μm) which contain dust deposits from regional sources and large-size particle dust (50–70 μm) produced by vehicles, people and livestock. The solid particles or droplets are accumulated on a surface due to gravity, electrostatic charge or other forces related to the fluid flow and heat transfer (for example, the thermophoresis force (Jiang and Lu 2015). The analysis shows that the dust with particles of a diameter smaller than 10 μm is not effectively removed even at a high airflow speed. Deposited particles are held at the near-surface region due to electrostatic forces, electrical potential, surface energy effects, capillary effects and the gravity. The structure of the surface and the surface roughness also play an important role and increase the surface friction. A detailed analysis of the particle deposition and the mechanics of contamination was performed by (Cuddihy 1983). He established that one of the most important processes was the cementing of the impurities in areas with a high-pollution level. The soluble and insoluble salts can be created in the air with a high humidity, which causes degradation of the PV module efficiency.

Studies demonstrated that large composition and dust distribution variation depends on specific local environmental conditions and location on Earth. In rural regions, the main source of the dust is the soil and plants while in the cities, contamination deposited on surfaces is the result of the dust and pollution which originates from different sources and which often contains organic compounds (Styszko et al. 2016; Szramowiat et al. 2016) and heavy metals (Styszko et al. 2015) derived from road transport and high emission from coal-fired heating systems (Samek et al. 2017). One identified a strong correlation between the quantity and structure of the dust and the season due to variation in the weather conditions (Sarver et al. 2013) and sources of pollution. Ta et al. demonstrate that more than 30% of the total annual quantity of dust is deposited in the spring months while in the winter season it is less than 20% (Ta et al. 2004). The dust samples from PV surfaces obtained from highly urbanised areas contain various compositions typical of the local area. The presence of cadmium, sulphur and antimony in the dust was detected by Fujiwara et al. (Fujiwara et al. 2011) while Bi et al. analysed the concentration of trace metals in different fractions of dust (Bi et al. 2013). All this depends on the dust composition and humidity. Moreover, undesirable cementing of the impurities may occur.

The influence of the dust deposition on the performance of photovoltaic modules is obvious but depending on the location, dust composition may be different and in consequence, the degree of reduction in the efficiency of PV modules may vary from location to location (Kaldellis and Kapsali 2011). However, usually 80% of the nominal module power is guaranteed by the solar panel manufacturer for a period of up to 25 years, the output power strongly depends on the environmental parameters and the ambient aggressiveness of the local area. It was observed (Rao et al. 2014) that dust deposition does not influence the open circuit voltage of a PV module while the short circuit current is significantly influenced by dust deposition and the drop in current output and as a consequence, the drop in generated power due to dust deposition constitutes an immense loss in energy produced and the economic loss of a PV power plant.

It was reported for the first time in 1942 that the mean reduction in incident solar radiation in the USA due to the dust effect is approximately 1% per month (Hottel and Woertz 1942). A large number of studies performed during the last decade demonstrated that dust accumulation on PV module surfaces may cause a significant decrease in solar conversion efficiency (Darwish et al. 2015). The study conducted in Málaga, Spain, (Zorrilla-Casanova et al. 2011) evaluated the average loss of energy from a PV module due to the dust effect to be about 4%, while in the long rainless periods, this value may rise as high as 20%. In contrast to this, a study performed in Dhahran, Saudi Arabia, indicates that a power decrease about 50% can occur in reference to PV modules when they are left uncleaned for 6 months (Adinoyi and Said 2013) while in CA, USA, a decrease is around 0.2% per day of rainless periods (Kimber et al. 2006). The maximum daily efficiency loss evaluated in Gdańsk, Poland, (a clean zone region) by Klugmann-Radziemska (Klugmann-Radziemska 2015), was about 0.8% and was significantly higher than the value reported for Spain, which was lower than 0.1% (Zorrilla-Casanova et al. 2011).

In the majority of cases, the natural cleaning of the photovoltaic modules surface due to heavy rain or snow melting is sufficient and performance is restored almost to the nominal level but this natural cleaning process can occur only in specific climatic conditions (typically with low or middle solar irradiation). However, if rain is desirable, light rain or water condensation together with impurities deposited on the surface may create an insoluble solid layer which is very difficult to remove. As it was also concluded by Klugmann-Radziemska (Klugmann-Radziemska 2015), the natural cleaning of th PV module surface in Gdańsk by rainfall, snow and the wind was not sufficient to recover nominal power, and frequent cleaning was strongly recommended. Mekhilef et al. concluded that dust, humidity and air velocity equally affect the performance of PV modules and that each of these components should not be considered separately (Mekhilef et al. 2012). On the other hand, Darwish et al. concluded that the dust pollution effect is local and is strongly linked to the pollution of the air in the area where the system is analysed (Darwish et al. 2015). For this reason, it is very difficult to develop a general model.

The characteristics of deposited dust and its impact on module efficiency are complex problems and it depends on specific local environmental conditions as well as climatic conditions which are site-specific factors. Important ambient conditions that affect dust characteristics include variation in wind velocity direction and magnitude, humidity, rain amount and instantaneous intensity and seasonal variations. Due to the fact that they are systematically studied in various locations of the Earth, the PV dust accumulation phenomena and the related power generation degradation can be better understood and addressed. In this research which was carried out in Kraków, Poland—one of the top ten polluted European cities—dust accumulation on the PV module front cover surface was studied by means of a systematic approach. The natural deposited dust density and the rate of dust accumulation under the local environmental conditions for several exposure periods were experimentally measured and analysed in a natural high-pollution city environment during the non-heating season. Additionally, determination of the natural cleaning process on the deposited dust was performed.

## Experimental set-up and methodology

### Site description and sample collection

Total suspended particles (TSP) were collected at the urban site, located in the centre of Kraków (50.066354 N, 19.918191 E) in the middle of a built-up area centre characterised by high traffic intensity and low air speed. A low volume sampler was placed on the roof of a five-storey building, next to the analysed PV modules. The sampler was equipped with a filter holder, needle valve, membrane pumps and gas meters. The sampler worked with an air flow of 1.4 m^3^ h^−1^. Samples were collected on quartz fibre filters (Pallflex, Pall Life Sciences) with a 47-mm diameter. Prior to sampling, the filters were thermally pre-cleaned at 550 °C for 5 h, cooled and equilibrated to a constant humidity. On weekdays, the filters were changed at 24 ± 2 h intervals, while during weekends (Friday, Saturday and Sunday), the filters remained in samplers for around 70 h. The sampled filters were equilibrated for 24 h to achieve conditions comparable with the conditions of the weighing of empty filters. The mass of the particulate matter was obtained as an average of the three subsequent weighing results of each filter. The OHAUS Discovery DV215CD balance with an accuracy of ± 0.01 mg was used for weighing.

The dust deposited on the PV module during variable exposure periods from 1 day up to 1 week was collected in plastic containers by means of a water collector, followed by freeze drying (Liophilizator Alpha 1-4 LD). The mass of accumulated dust was determined gravimetrically, as a difference of mass plastic containers before and after sampling.

### Photovoltaic modules

**Fig. 1:**
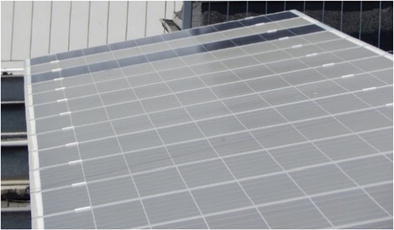
An experimental set-up for in situ natural dust deposition on the photovoltaic modules

In this experiment, nine identical Sharp ND-RJ260-type polycrystalline photovoltaic modules with nominal peak power P_PV_ = 260 W and a 1.6-m^2^ front surface glass area (excluding aluminium frame) were used. The modules featured a tilt angle *β* = 15^o^, and an azimuth *γ* = 20^o^ West, where tilt is the angle in degrees from the horizontal. One should note that the analysed PV system was in normal operation. One of the modules was used to collect daily samples (every 24 h) while dust was collected regularly from other modules, after 2, 3, 4 days and after 1 week of exposition. Two of them was not cleaned at all and left dusty for the photovoltaic modules efficiency analysis (Jaszczur et al. 2017). The samples were collected in spring during the non-heating season in a period of 5 weeks (11.05–13.06.2017). An experimental set-up for in situ natural dust deposition on the photovoltaic modules is shown in (Fig. 1).

### Meteorological data

The summary for meteorological data (air temperature, wind speed, wind direction, humidity and precipitation volume) which have been recorded during the study is presented in (Table 1). Data includes the mean values and the range of the temperature, relative humidity and wind velocity. The weather data of atmospheric conditions in Kraków were obtained from the Faculty of Physics and Applied Computer Science Vaisala WXT520 automatic meteorological station. The station was placed on the roof near the building where the dust samples and total suspended particles have been collected. The concentration of PM10 was obtained from the Voivodeship Inspectorate of Environmental Protection in Kraków at an urban background station (Kurdwanów, Złoty Róg and al.Krasińskiego Stations).

**Table 1:** The summary of weather conditions during the study period

Sampling period	11.05–13.06.2017
Mean temperature (Min–max temp.) (^o^C)	16.81 (5.72–22.35)
Humidity (%)	55.38 (39.94–80.13)
Precipitation volume (mm)	0.45 (0–3.03)
Wind velocity (m s^−1^)	1.92 (1.12–3.47)

## Results and discussion

### Total particulate matter (TSP) and PM10

**Fig. 2:**
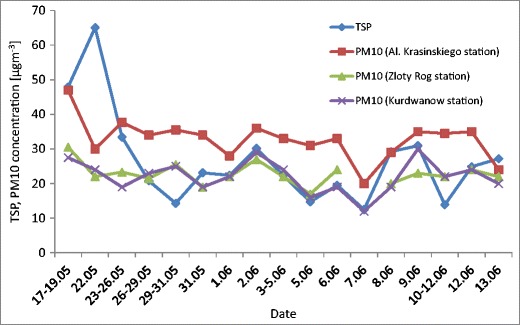
Variations of the total TSP and PM10 particulate matter concentrations during the study period

The concentration of total suspended particles in the air during the study period ranged from 12.5 to 65.1 μg m^−3^ and the weight average value TSP was about 26.6 μg m^−3^. The concentration of TSP for PV module location was compared to the concentration of PM10 noted by the Voivodeship Inspectorate of Environmental Protection in Kraków at an urban background station (Kurdwanów and Złoty Róg Stations). The concentration of PM10 recorded at both stations was very similar. The mean PM10 concentration was 24.2 μg m^−3^ and the concentration varied in the range 12.0–40.0 μg m^−3^. The variations of the PM10 and TSP concentrations during the period are presented in (Fig. 2). Generally speaking, PM10 was responsible for 82% of the TSP mass. In some cases, PM10 concentrations were higher than TSP concentrations, which could be the result of the different locations for measurement locations. The Kurdwanów station is located about 8 km and the Złoty Róg station about 3 km from the TSP sampling location. The third PM10 station (al. Krasińskiego station) is located nearest, about 1 km from TSP sampling station. Concentrations of PM10 at the al. Krasińskiego station is higher than concentrations of TSP almost over the entire measurement period. The reason for this may be not the horizontal but the vertical distance. It should be noted that the concentration of PM10 at the al. Krasińskiego station is measured at a ground level which is significantly lower (about 20 m) than the TSP measurement. Additionally, this PM10 station is located between two main city arteries characterised by the highest traffic intensity. The amount of dust deposited on PV modules could depend on the height of their location.

### Particle deposition density and meteorological data

**Fig. 3:**
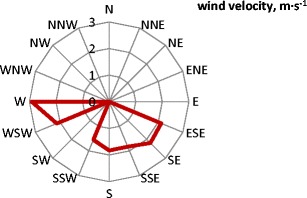
Wind velocity and directions during the study period

In the sampling period, temperature varied from 5.7 to 22.3 °C, humidity from 40 to 80% and precipitation volume was up to 6 mm with a maximum duration of 186.4 min. The air velocity and direction from the meteorological station located nearby is shown in (Fig. 3). The dominant wind direction that was observed in the sampling period was the West-southwest, WSW direction which means that the wind direction is in good relation to the module front surface (tilt angle *β* = 15^o^, azimuth *γ* = 20^o^) and that the wind will cause natural surface cleaning as well as module cooling. During the study period, the wind velocity varied between 1.1 and 3.4 m s^−1^.

**Fig. 4:**
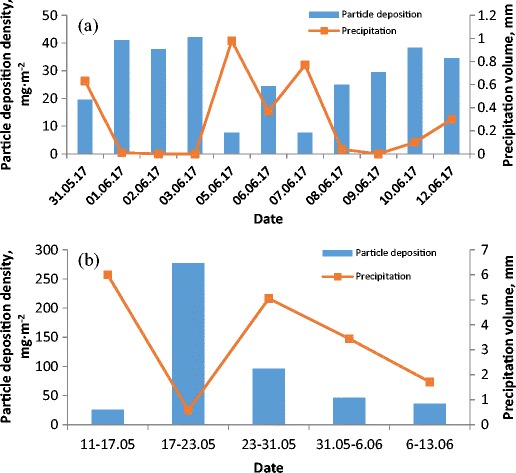
Variations of particle deposition density and precipitation during the study period, for one-day (**a**) and one-week (**b**) measurement

Figure 4a, b shows the particle deposition densities on identical photovoltaic modules depending on the duration of deposition which varied from 1 day up to 1 week. Particle deposition densities are strongly influenced by precipitations which varied day to day in a number of precipitations as well as in duration. The maximum particle deposition densities for one-day measurements amounted to 42.1 mg m^−2^ (see Fig. 4a) and 277 mg m^−2^ for a one-week measurement (see Fig. 4b). The difference between the highest and lowest recorded density was about 34.5 and 251.2 mg m^−2^ for 1-day and 1-week samples, respectively and depended on the precipitation intensity

The highest particle deposition densities were observed on days 01.06–03.06 and the week 17–23.05 when no rainfall occurred (see Fig. 4 and b). One-day particle deposition densities in these days were very stable and varied only from 37.7 to 42.1 mg m^−2^.

It is clear that precipitation (intensity and duration) has a significant influence on air pollution (compare Figs. 3 and 4) and, eventually, on dust deposition. The rainfall also cleared the modules directly. The lowest TSP and PM10 were recorded after the rain 12.51–14.73 μg m^-3^and what is significant, in order to clear the air from pollution (represented by TSP) and the modules from dust (represented by particle deposition densities) a certain threshold of precipitation is required. This is particularly observable for the days 10–11.06 when small rainfall occurs which is sufficient to clean the air (one of the lowest TSP 13.87) but insufficient to clean the modules (particle deposition densities high and increases). On the next day—12.06—the level of precipitation was sufficient to decrease particle deposition densities in reference to the previous day.

The maximum intensity of precipitation was 22.03 mm h^−1^ (maximum peak intensity 62.0 mm h^−1^) and was the highest for 31.05–06.06. The maximum precipitation intensity for 23–31.05 and 06–13.06 amounted to 10.44 mm h^−1^ (maximum peak intensity 20.5 mm h^−1^) and 6.98 mm h^−1^ (maximum peak intensity 29.0 mm h^−1^), respectively.

However, on some days 08–09.06 no rainfall occurred and the highest TSP concentrations were observed but particle deposition densities were between 24.9 and 29.5 mg m^−2^, respectively.

**Fig. 5:**
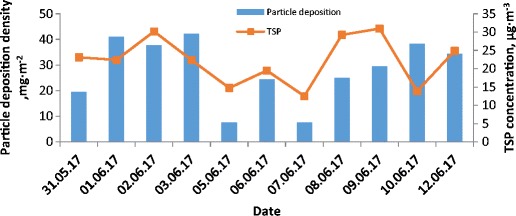
Variations of particle deposition density and TSP concentration versus time for 1-day measurements

**Fig. 6:**
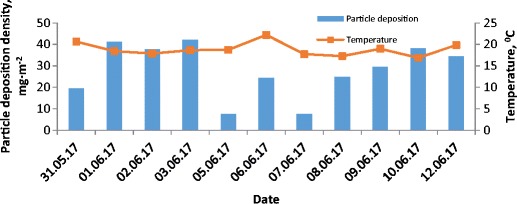
Variations of particle deposition density and temperature versus time for 1-day measurements

**Fig. 7:**
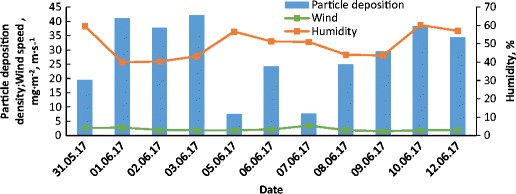
Variations of particle deposition density and humidity versus time for 1-day measurements

In (Figs. 5, 6 and 7), the variations of particle deposition density together with TSP concentration, temperature or humidity during the study period for one-day measurements are shown.

The low particle deposition density recorded on 08–09.06 for very high total suspended particle concentration in the air (above 30 μg m^−3^) can be correlated with the very low air humidity of about 40% (see Figs. 5, 6 and 7 for reference). The highest growth of particle deposition density is related to the highest growth of total suspended particle concentration in the air from 12.5 to 29.2 μg m^−3^ and was observed at 08.06, after very intense rainfall which occurred on 07.06.

The growth of particle deposition density in the subsequent days 09–10.06 was much lower and was 4.5 and 8.7 mg m^−2^, respectively in reference to the deposition density on 8.06. This increase, particularly on the 10.06, is unexpected because of the extremely low level of total suspended particle concentration in the air of about 13.87 mg but on that day the highest humidity was recorded, reaching up to 60%. For the environmental conditions observed in those days the growth of humidity increased particle deposition density. It could be assumed that precipitation episodes and its intensity but also the humidity level plays a key role in dust particle deposition.

**Fig. 8:**
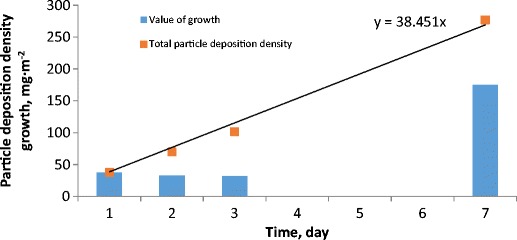
Particle deposition density and growth of particle deposition density in subsequent days after excluding rainfall days

In (Fig. 8) the particle deposition density and the growth of particle deposition density on the surface of the photovoltaic module (front cover) in subsequent days excluding rainfall days are shown. It can be seen that the particle deposition density increases systematically with time. This increase for up to 7 days of detailed analysis (longer period was not possible due to rainfall which occurs almost every week) is linear. The highest increase, 40.3 mg m^−2^ was noted on the first day of dust accumulation on the module surface. The daily increases in two subsequent days were lower and varied between 29.5 and 32 mg m^−2^. On the subsequent days—between the fourth and seventh day—the increase was 175.1 mg m^−2^, which could suggest that daily growth amounted to 43.8 mg m^−2^. One could interpret this in the following way: the daily growth up to a week is well correlated with linear growth. However, more experiments and tests for longer exposure periods have to be conducted to see if and when this growth starts to deviate from linear growth.

**Fig. 9:**
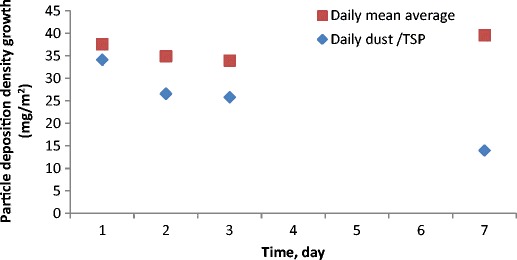
The daily averaged particle deposition and normalised daily average particle deposition based on 1, 2, 3 and 7-day measurement

The daily averaged particle deposition and normalised daily average particle deposition is presented in (Fig. 9). The experimentally evaluated particle deposition density for 1, 2, 3 and 7-day analysis was divided by the number of days. One may see that the increasing deposition period for the daily average particle deposition decreases. The only exception to this trend can be observed for the last point (based on deposition of 7 days) for which daily average particle deposition increases. The reason could be associated with very high TSP during 7-day deposition. In order to account for this effect normalised daily average particle deposition was calculated where the normalisation parameter was the mean TSP value during dust deposition. As one may see, the normalised value for 7-day deposition is now much lower and a declining trend can be observed.

### Particle dry deposition fluxes

**Fig. 10:**
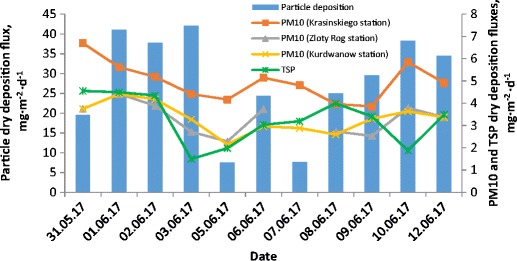
Variations of particle dry deposition fluxes obtained for daily samples

TSP dry deposition fluxes (I_A_), expressed as mg of TSP per m^2^ and per day, were calculated according to the procedure applied in the previous study by (Styszko et al. 2015). The daily average particle dry deposition fluxes obtained for PM10 observed on different measurement stations, TSP concentrations and measured dust deposition on PV module surface are shown in (Fig. 10).

It could be stated that the daily dry deposition fluxes on module surface follow the same trend as the fluxes obtained for PM10 and TSP. However, the values of daily fluxes noted for PV modules were at least two times higher. It could be expected that additional forces, for example, electrostatic forces, play a significant role in dust deposition on the surface. In order to evaluate this phenomenon and to evaluate the correlation between all environmental components, more experiments under various natural environmental conditions have to be performed.

## Conclusions

Many environmental parameters affect the energy production from photovoltaic modules and dust could be one of the main reasons for its degradation. The dust, which represents a mixture of different pollutants, is determined by the geographical site. Important ambient conditions that affect dust characteristics are variation in wind velocity, direction and magnitude, humidity, rain amount and instantaneous intensity and seasonal variations. Due to the fact that they are systematically studied in various locations of the Earth, the PV dust accumulation phenomena can be better understood and addressed. This study was performed in order to investigate the natural dust accumulation process on the front cover glass of PV modules during the non-heating season in one of the most polluted European cities, Kraków, in different meteorological conditions. The time-dependent particle deposition and their correlation to the air pollution with particulate matter were analysed. The data contained in this paper will be useful for prediction of dust deposition on the basis of environmental conditions which are of interest to system developers and operators. The major findings can be summarised as follows:

- The measured daily particle deposition densities range from 7.5 to 42.1 mg m^−2^.
- The measured weekly particle deposition densities range from 25.8 to 277.0 mg m^−2^.
- Precipitation volume and its intensity (and what is also important maximum peak intensity), as well as humidity, significantly influence the particle deposition density.
- The rate of dust accumulation reaches approximately 40 mg m^−2^ day^−1^ in the no-precipitation period and was at least two times higher than fluxes calculated on the basis of PM10 and TSP concentrations. It seems that other forces such as electricity affect the influence of module surface on dust deposition.
- The rate of dust accumulation was the highest for short exposure times and decreases when the period increases.
- For the cases of photovoltaic modules, a Sharp ND-RJ260-type device installed with a tilt angle *β* = 15^o^ was used; the dust deposited during 1 week of no-precipitation period was significant but the natural cleaning process by rainfall and the wind was sufficient to remove most of the “fresh” dust (more than 90%) as well as to clean other kinds of impurities from the surface. This was particularly the case when the maximum value for the intensity of precipitation was higher than 6.98 mm h^−1^ and when the peak intensity of precipitation reached at least 16.8 mm h^−1^). The values lower than the ones that were presented are not sufficient to clean the modules efficiently or they even create a layer of dust which is difficult to remove.
